# Towards Semantic Sensor Data: An Ontology Approach

**DOI:** 10.3390/s19051193

**Published:** 2019-03-08

**Authors:** Jin Liu, Yunhui Li, Xiaohu Tian, Arun Kumar Sangaiah, Jin Wang

**Affiliations:** 1 College of Information Engineering, Shanghai Maritime University, Shanghai 201306, China; jinliu@shmtu.edu.cn (J.L.); yunhuili@shmtu.edu.cn (Y.L.); xhtian@shmtu.edu.cn (X.T.); 2 School of Computing Science and Engineering, Vellore Institute of Technology, Tamil Nadu 632014, India; arunkumarsangaiah@gmail.com; 3 School of Computer & Communication Engineering, Changsha University of Science & Technology, Changsha 410114, China; 4 School of Information Science and Engineering, Fujian University of Technology, Fuzhou 350108, China

**Keywords:** sensor data, domain ontology, domain ontology mapping, ontology-based data fusion

## Abstract

In order to optimize intelligent applications driven by various sensors, it is vital to properly interpret and reuse sensor data from different domains. The construction of semantic maps which illustrate the relationship between heterogeneous domain ontologies plays an important role in knowledge reuse. However, most mapping methods in the literature use the literal meaning of each concept and instance in the ontology to obtain semantic similarity. This is especially the case for domain ontologies which are built for applications with sensor data. At the instance level, there is seldom work to utilize data of the sensor instances when constructing the ontologies’ mapping relationship. To alleviate this problem, in this paper, we propose a novel mechanism to achieve the association between sensor data and domain ontology. In our approach, we first classify the sensor data by making them as SSN (Semantic Sensor Network) ontology instances, and map the corresponding instances to the concepts in the domain ontology. Secondly, a multi-strategy similarity calculation method is used to evaluate the similarity of the concept pairs between the domain ontologies at multiple levels. Finally, the set of concept pairs with a high similarity is selected by the analytic hierarchy process to construct the mapping relationship between the domain ontologies, and then the correlation between sensor data and domain ontologies are constructed. Using the method presented in this paper, we perform sensor data correlation experiments with a simulator for a real world scenario. By comparison to other methods, the experimental results confirm the effectiveness of the proposed approach.

## 1. Introduction

Recently, various intelligent Internet of Things (IoT) based algorithms [1] and applications [2] have been developed by making use of large amount of sensor data, for example, mobile data reception in wireless sensor networks [3], and various applications in urban sustainable development [4]. To optimize the utilization of data from multiple sources for decision making, meaningful sensor data should be achieved [5,6]. Building sensor ontology and mapping sensor data to domain ontology provides a solid foundation for sensor data sharing, reuse and fusion in a variety of IoT applications [7,8,9]. Further, semantic sensor networks (SSN) are proposed to formally express semantic associations with an existing ontology [10]. However, due to the diversity of the domain ontologies and the ontology construction methods, the domain ontologies often have significant differences. In order to dig out more rules or knowledge with multiple existing heterogeneous ontologies, we need to establish mapping relationships among the ontologies. Therefore, it is critical to study how best to perform ontology mapping in order to associate multiple domain ontologies with the presence of sensor data.

Many researchers have done a lot of work on ontology related areas. In the early days, domain experts used manual methods to establish ontology mapping. Since then, these methods have evolved to incorporate semi-automated and automated methods. At present, there are three main types of ontology mapping methods: machine learning based, similarity calculation based and background knowledge based. The machine learning-based mapping method can be regarded as a model for information classification, where the information in ontology is used to predict the objects that each concept may map to. However, such methods do not fully utilize the information in the ontology. The mapping method based on background knowledge relies on the existing domain knowledge base. This kind of method is more accurate, however it’s efficiency and recall rate remains insufficient. Similarity-based mapping methods are generally based on a similarity calculation of concepts in different ontologies which may have been built with different methods, thus the application field of this strategy is narrow. To overcome the deficiencies of existing methods, we proposed a novel similarity evaluation method which utilizes multiple strategies to establish the relationship between domain ontologies and uses a random forest algorithm to perform the classification of instances in order to make better use of sensor data and reduce manual intervention. In addition, this method can reduce calculation efforts that are not critical in the analytic hierarchy process and improve the computation efficiency in the case of a large volume of data.

The remainder of this article is structured as follows. In Section 2, we introduce the related applications of sensor ontology in the field of IoT and the research work related to ontology mapping. Based on the analysis of the sensor data processing method and the ontology correlation method in the literature, we propose a random forest-based method to classify sensor instances in Section 3. Then, in Section 4, we propose a multi-strategy similarity calculation method utilizing the sensor data classification result to estimate the similarity between ontology concepts. In Section 5, the experimental results of the proposed method are presented. Finally, in Section 6, some conclusions are drawn. 

## 2. Related Work

In order to improve human-computer interaction, ontology is used to solve the problem that one concept may correspond to multiple words. Domain ontology is a professional knowledge base which describes the relationship between concepts within a specific field. For example, in the field of IoT, there has been a lot of work on the interoperability of the IoT. These include many European projects such as FIESTA-IoT, Inter-IoT, and LOV4IoT, etc. In the field of sensors, there are 12 main categories of well accepted sensor ontologies [11]. Based on the wireless sensor network composed of these sensor ontologies, there has been a significant amount of research in a variety of fields such as network energy saving [12], collaborative computing [13], network routing [14] and so on. Although these sensor ontologies are constructed according to the continuously improved unified ontology framework SSN/SOSA, there are non-uniform definitions of the same concepts in different application fields, which make these sensor ontologies difficult to share and reuse [15].

Although there are already a variety of ontology construction methods in each specific domain, the ontology in these specific fields is difficult to expand and apply to other domains. This requires the reuse of the ontology and the association between the ontologies, and the establishment of the relationship between the ontologies in different domains. Liu et al. [16] proposed a construction method for a multi-domain ontology that can be used for large-scale unstructured text. This method is effectively applied to the construction of a multi-domain ontology in the shipping industry. Ehrig et al. [17] correlate ontology by comparing similarities between entities in different ontologies. Mao et al. [18] propose the use of quaternions (Entity 1, Entity 2, Relation, Confidence) to represent ontology relations. In addition, in order to solve the problem of semantic heterogeneity, some research work is carried out around ontology matching. Ontology matching enables the knowledge and data expressed in the ontology to interoperate by studying the semantic relationships between the corresponding entities and then applying them to various tasks. Otero-Cerdeira et al. [19] proposed an ontology matching method based on the context data collected by the sensor, and deployed it in a smart city to improve the interoperability of information. Fernandez et al. [20] presented a system for ontology alignment in the semantic sensor web which uses fuzzy logic techniques in order to combine similarity measures between entities of different ontologies. Their similarity evaluation strategy mainly consists of the context-related semantic similarity degree of the entity name and the degree of structural similarity of the ontology concept. In order to implement ontology matching on the semantic web, there are some methods [21] that combine deep learning techniques. They developed a system that employs learning techniques to semi-automatically create semantic mappings between ontologies.

Ontology integration [22] refers to the process of establishing a mapping among entities, processing mappings, and aligning or merging two or more ontologies into a “new” ontology. Ontology integration is mainly used to solve two types of problems: (1) improve and enrich the existing ontology content and structure, and reuse the existing ontology; (2) solve the problem of heterogeneous information among the applications of different fields. Based on the different degree of ontology integration [23], the ontology integration can be divided into three categories: ontology mapping, ontology alliance and ontology merged. The degree of integration is strengthened in turn. Ontology mapping has various applications, from machine learning, concept lattice, and formal theories to heuristics, database schema and linguistics. The practice of ontology mapping ranges from academic prototypes to large-scale industrial applications [24]. Research on ontology mapping needs to study ontology feature representation and extraction. For feature extraction, Zeng [25] proposed a method to learn features for distant supervised relation extraction (DSRE) using a method of generative adversarial networks (GANs). This approach extracts more efficient feature representations than other neural network models. Similar work by using GANs on digital signal modulation classification can be found in [26].

The objective of ontology mapping is to find correspondences in entities from multiple ontologies [27]. It is an effective way to address knowledge sharing and the reuse of heterogeneous ontologies in semantic webs, which solves the exchange of complex information [28]. The method of ontology mapping can be divided into the four categories. Firstly, statistical-based ontology mapping in which a statistical approach is used in the mapping process. Jung M [29] proposed a method based on Bayesian network, while Swat [30] proposed a method based on probability distribution in the mapping process. Secondly, there is rule-based ontology mapping in which the heuristic rules are given by domain experts during the mapping process. The mapping method proposed by Ehrig et al. [17] is based on heuristic rules. This method first denotes the heuristic rules by domain experts and calculates the similarity of each pair of entities to obtain the calculated results. Thirdly, there is ontology mapping based on machine learning. Moran et al. [31] propose an ontology-based classification method using the decision tree classifier method for multi-source classification of nature conservation areas. Finally, an ontology mapping method based on the ontology concept feature calculates the similarity from the different aspects of the concept name, the instance of the concept, the attribute of the concept and the structure of the ontology. In addition, there are some studies on ontology feature mapping. Ravikumar [32] used deep learning methods to extract features and then used binary tree support vectors for feature classification. This method shows that the problem of feature mapping can be explored by using feature classification. Liu [33] proposed a new way to mark entity categories, using neural network models to extract multiple relationships. This method has a good effect on describing complex mapping relationships and extracting mapping relationships. 

According to types of the objects that are chosen to construct the mapping relationship, we can also classify ontology mapping into the following three categories: (1) mapping between an integrated global ontology and local ontologies, (2) mapping among local ontologies, and (3) mapping on ontology merging and alignment [34]. The first category of ontology mapping supports ontological integration by describing the relationship between an integrated global ontology and local ontologies. This category supports ontology integration processes. Methodological aspects of ontology integration relate to how this mapping is denoted [35]. This mapping specifies how concepts in global and local ontologies map to each other, how they can be expressed based on queries, and how they are typically modeled as views or queries [36]. The second category enables interoperability for highly dynamic and distributed environments as a form of mediation among distributed data in such environments. This category provides interoperability for highly dynamic, open and distributed environments and can be used for mediation among distributed data in such environments [37]. The third category is used as a part of ontology merging or alignment as an ontology reuse process. In this case, ontology mapping establishes a correspondence among source (local) ontologies to be merged or aligned, and determines the set of overlapping concepts, synonyms, or unique concepts to those sources [38]. This mapping identifies similarities and conflicts among the various source (local) ontologies to be merged or aligned [39].

Related ontologies have semantic relationships between similar entities of two different ontologies. This kind of association lays an important foundation for semantic sensor networks. Considering the problem of semantic association, Wang [40] proposes a semi-structured and self-describing Extensible Markup Language (XML) data organization form, which realized the model of solving semantic association problems through semantic dependence in the process of data integration. Xiong [41] proposed a new deep learning model based on the Continuous Bag of Words (CBOW) model [42] and Convolutional Neural Networks (CNNs). This model uses a distributed vector representation to realize the semantic association between large amounts of data in the dataset, with semantic relativity and accuracy.

Ontology association also supports the semantic query of multiple ontologies from the perspective of information retrieval. In addition, some researchers have used machine learning or heuristic rules in order to find specific mapping patterns [43], and some have resolved ontology mapping by analyzing the semantic information of elements in the ontology [44,45]. Pinkel et al. [46] presented a new version of Relational-to-Ontology Data Integration (RODI), which significantly extends the previous benchmark, and they use it to evaluate various systems. RODI includes test scenarios from the domains of scientific conferences, geographical data, and oil and gas exploration. Scenarios are constituted of databases, ontologies, and queries to test the expected results. Systems that compute relational-to-ontology mappings can be evaluated using RODI by checking how well they can handle various features of relational schemas and ontologies, and how well the computed mappings work for query answering. Forsati et al. [47] formalized ontology mapping in heterogeneous knowledge bases as an optimization problem, and an efficient method called harmony search based ontology mapping (HSOMap) was proposed, that effectively finds a near-optimal mapping for two input ontologies. Helou et al. [48] presented a large-scale study on the effectiveness of automatic translations to support two key cross-lingual ontology mapping tasks: the retrieval of candidate matches and the selection of the correct matches for inclusion in the final alignment. Thoroughly discussing several findings of the research, which are believed to be helpful for the design of more sophisticated cross-lingual mapping algorithms.

As mentioned above, in terms of ontology-based sensor data processing, there is a lack of a universal efficient domain ontology mapping method. In addition, for the association method between ontologies, most of the research work mainly match literal meanings or calculate the similarity of concept names. How to reduce the semantic conflict and human intervention to realize the semi-automatic or automatic ontology mapping is still a challenging task in the field of ontology mapping.

## 3. Instance Classification

The sensor can collect a series of data including location, temperature, wind speed, altitude, humidity and other attributes. However, in different ontology structures, the same sensor instance can be divided into different sets of concepts. For example, for different shipping bodies, there are two main ways to divide the concept of containers: (1) dry container, bulk container, liquid cargo container, reefer container, and special container, such as automobile container, animal husbandry container, animal skin container, etc.; and (2) reefer container, dress hanger container, open top container, flat rack container, tank container, reefer container, platform container, ventilated container, insulated container, etc. It is not difficult to see that the concept of the animal husbandry container and the ventilated container in the above division has a certain degree of an overlapping relationship. Suppose a series of data collected by the sensor is expressed as $R=\left\{r_{1},r_{2},\ldots r_{n}\right\}$, where $r_{i}\left(1\leq i\leq n\right)$ represents the data collected on the $i_{th}$ attribute. Then, as far as the temperature concept of the container is concerned, according to the result f(R) of the attribute data set R in the sensor instance for each concept in the ontology, we can use the sensor instance as an example of the temperature concept in the animal husbandry container, or as an example of the temperature concept in the ventilated container.

The above situation is widely presented in the ontology of different structures. According to the features of sensor instance data, relationships that exists between different sets of the sensor instance data can be used a measure for the similarity between concept pairs in the ontology. In our method, a random forest algorithm, denoted as f, is used to classify sensor instances into different concept sets by using various attribute values in the sensor data as the basis for classification. When we use random forests to build a dataset for a sensor, we use the attribute set R of all sensors as a set of attributes for each sensor’s data. Assume that there is a total of M sensor data. For a specific sensor, the uncollected attributes are recorded as default values. This process ensures that all sensor data has a uniform dimension. In addition, for the concept, we use a manual labeling method to mark a part of the data, which is denoted as Y. This data set consists of the sensor’s various attribute values X and concept tags Y. It is important to note that we deal with the discrete attribute values by transforming the expert definitions into numerical form.

We denote the training data set as D, which needs to be divided into K classes. According to the calculation of information gain, we select the attribute A in sensor data as the basis of decision division. Then the information gain can be defined as follows:

(1) g(D,A)=H(D)−H(D|A).

where H(D) represents the empirical entropy of and H(D|A) represents the empirical conditional entropy of selected A.

Based on this, we build a decision tree. Each non-leaf node in the decision tree represents a test on a feature attribute. Each branch represents a decision condition that the data is satisfied. Each leaf node represents a category to which the data ultimately corresponds. The following Algorithm 1 shows the process of generating an unpruned decision tree for uncategorized sensor data.

Next, we need to prune the generated decision tree, cut off some unnecessary branches, and control the complexity of the decision tree by adding regular terms. Definition C(T) represents the prediction error of the model for the training data. |T| represents the complexity of the model, which is the number of leaf nodes. The parameter α balances the training error and the model complexity. The loss of the decision tree is expressed as follows:

(2) $$C_{\alpha }\left(T\right)=C\left(T\right)+\alpha \left|T\right|.$$

**Algorithm 1 Decision tree generation algorithm**
**Construct training set from sensor data**X is a matrix of R×M, $X_{ij}$ represents the *j*-th feature of the *i*-th sample.Y is a matrix of R×1, $Y_{i}$ denotes the class label of the *i*-th sample.**Build a decision tree**
   **If** all the sample values of X are the same, or all the class labels of Y are the same, or R<2, a leaf node is generated, and the class of this node is the class of the most number in X.   **else:**
    Select m randomly from M features.
    Among these m features, the maximum information gain is denoted as p.
    **If:** the value of feature p is discontinuous
     V is any value of p
     $X_{V}$ is used to represent the sample whose feature p takes V, $Y_{V}$ is the corresponding class.
     $Childv=Generate\left(X_{V},Y_{V}\right)$
     **Return** a decision tree node
    **If:** the value of feature p is continuous
     *t* is the best split threshold.
     **If:**
$X_{LO}$ represents a sample set whose values of feature p is less than t, and $Y_{LO}$ is its corresponding class.
     $Childlo=Generate\left(X_{LO},Y_{LO}\right)$
     **If:**
$X_{HI}$ represents a sample set whose values of feature p is greater than or equal to t, and $Y_{HI}$ is its corresponding class.
       $Childlo=Generate\left(X_{HI},Y_{HI}\right)$
     **Return** a decision tree node

The pruning process is shown in Algorithm 2. By generating a large number of decision trees, these decision trees are combined to build a random forest model. The random forest training algorithm is shown in Algorithm 3. And random forest classification algorithm is shown in Algorithm 4.


**Algorithm 2 Pruning algorithm**
**1.** Calculate the information gain of each node. **2.** Recursively upwardly from the leaf node of the tree, calculate the loss of the leaf node before and after the parent node: $C_{\alpha }\left(TB\right)$ and $C_{\alpha }\left(TA\right)$. **If**
$C_{\alpha }\left(TA\right)<C_{\alpha }\left(TB\right)$: Prune. **3.** Repeat step 2 until it cannot continue.


**Algorithm 3 Training algorithm**
**1.  Construct set from sensor data**: Given training set S, test set T, feature dimension F.
  **Determine the parameters**: The number of decision trees t, the depth of each tree d, and the number of features f used by each node.
  **Termination conditions**: The minimum number of samples on the node S, the minimum information gain on the node m.
**2.** From S, there is a training set S(i) of the same size as the extracted size S, as a sample of the root node, and training is started from the root node.
**3.**
**If**: the termination condition is reached on the current node,
   Set the current node as a leaf node.
  **If**: the current node does not reach the termination condition,
   The f dimensional features are randomly selected from the F dimensional features without replacement. Using this f dimensional feature, find the best one-dimensional feature k and its threshold th.
   The sample whose k dimension feature is less than th at the current node is divided into left nodes, and the rest is divided into right nodes.
   Continue to train other nodes.
**4. Repeat** 2, 3 until all nodes have been trained or marked as leaf nodes.
**5. Repeat** 2, 3, 4 until all decision trees have been trained.


**Algorithm 4 Random forest classification algorithm**

Starting from the root node of the current tree, according to the current node’s threshold th, it is determined whether to enter the left node (<th) or enter the right node (≥th) until a certain leaf node is reached and the predicted value is output.Repeat 1 until all t-trees have output predictions. This will give a class with the largest sum of predicted probabilities in all trees.


## 4. Associating Domain Ontology Based on Sensor Instances

In this section, we present a novel domain ontology mapping method. A higher similarity between the ontologies implies a stronger equivalence relation. In our method, we will use three similarity calculation strategies in order to assess the similarity of concepts between ontologies, and use the analytic hierarchy process to construct mapping rules between different concepts of domain ontology.

### 4.1. Semantic Strategy

For one concept pair $W_{1},W_{2}$ in the ontology $O_{1},O_{2}$, if they are consisted by the same or similar characters, it can be confirmed that the concept pair $W_{1},W_{2}$ has the same or similar meaning. In the similarity analysis of the concept pairs, we find that it is a better strategy to evaluate the semantic similarity based on the knowledge base, HowNet [49]. There are more than 173,000 words in HowNet which are described by bilingual DEF. Different DEF descriptions are used to express the different semantics of a word. DEF is defined by a number of sememes and the descriptions of semantic relations between words. It is worthy to mention that a sememe is the most basic and the smallest unit which cannot be easily divided, and the sememes are extracted from about six thousand Chinese characters.

According to HowNet, we describe concept pairs separately through sememes. Then we denote the concept similarity based on sememes described by the positional relationship of the sememe hierarchy tree. $Sim(W_{1},W_{2})$ represents the semantic similarity between $W_{1}$ and $W_{2}$ in the ontology.

For semantic similarity, we use the sememe distance and the sememe depth to calculate. Among them, the meaning of the sememe distance is the length of the path from sememe feature $p_{1}$ to sememe feature $p_{2}$ in the same sememe hierarchy tree, which is denoted by $Dist(p_{1},p_{2})$. If the sememe features $p_{1}$ and $p_{2}$ are not in the same sememe hierarchy tree, then we set $Dist(p_{1},p_{2})$ to a fixed value of 20.

Sememe depth refers to the path length from the root node on the sememe hierarchy tree to this p node, denoted by dep(p).

The semantic similarity calculation combining the sememe distance and the sememe depth is expressed as:

(3) $$Sim(p_{1},p_{2})=\frac{\omega (dep(p_{1})+dep(p_{2}))+\theta }{Dist{\left(p_{1},p_{2}\right)}^{2}+\omega (dep(p_{1})+dep(p_{2}))+\theta }.$$

Among them, $dep(p_{1})$ and $dep(p_{2})$ represent the sememe depths of $p_{1}$ and $p_{2}$. ω is an adjustable parameter, which is the sememe path length when the sememe similarity is equal to 0.5. θ is also an adjustable parameter.

Equation (3) highlights the degree to which sememe distance affects overall similarity assessment. This is because when the sememe distance is large, the corresponding similarity is low; but when the sememe distance is small, this means that the two concepts are similar. Our formula highlights the role of sememe distance.

In addition, we also consider the effect of sememe distance on similarity calculation. For two sememes, the similarity of sememes decreases as the level difference increases. The more similar two sememes are, the smaller the level difference. We use the level differences in the sememe tree to represent the semantic differences in concepts. In the formula, we use the parameter ω to add the sememe distance information to the similarity calculation.

The use of the tunable parameter θ limits the semantic similarity $Sim(p_{1},p_{2})$ from *0* to *1*. Our formula takes into account the influence of the sememe level depth and the sememe distance on the similarity, and at the same time gives the appropriate constraints on the similarity. Therefore, reasonable results can be obtained.

In the description of a sememe, a feature structure will include multiple features, but the first sememe description is more important than others. Therefore, when calculating sememe-based semantic similarity, we give different weights for sememes in different positions in order, and ensure that the first sememe description has the highest impact weight. Thus, we combine all the similarities of the sememe calculations as:

(4) $$Sim_{pri}(W_{1},W_{2})=\sum_{i=1}^{N}\lambda_{i}\prod_{j=1}^{i}Sim_{j}(W_{1},W_{2}).$$

where $\lambda_{i}\left(1\leq i\leq N\right),(\lambda_{1}\geq \lambda_{2}\geq \cdots \geq \lambda_{N})$ represents the calculation weights of N original features and $\sum_{i=1}^{N}\lambda_{i}=1$. $Sim_{j}(W_{1},W_{2})$ calculates the semantic similarity of the jth sememe feature according to the above formula.

### 4.2. Instance Strategy

We believe that the similarity between two concepts can be reflected by the relationship among the collection of concept instances. The collection of instances contains the specific semantic relations to a certain extent. We denote the concept instance similarity as $Sim_{ins}(W_{1},W_{2})$. The main idea of using a concept-based calculation method is to measure the ratio of the total number of instances in the intersection among the set of instances. 

We set a threshold to measure the similarity of concepts to $W_{1},W_{2}$ which represent the concept pair in ontology $O_{1},O_{2}$. $U^{W_{1}},U^{W_{2}}$ indicate the set of instances for the concepts $W_{1},W_{2}$. $\left|U^{W_{1}}\right|,\left|U^{W_{2}}\right|$ represent the number of instances in the corresponding instance set. In addition, we assume that $U_{1},U_{2}$ are the set of instances corresponding to the ontology $O_{1},O_{2}$. $U_{1}^{W_{1},W_{2}}$ means that in the ontology $O_{1}$, it belongs to both concept $W_{1}$ in the ontology $O_{1}$ and concept $W_{2}$ in the ontology $O_{2}$. $\left|U_{1}^{W_{1},W_{2}}\right|$ represents the number of instances in the ontology $O_{1}$. $U_{2}^{W_{1},W_{2}}$ and $\left|U_{2}^{W_{1},W_{2}}\right|$ are similar to the above.

For the instance set $U_{1},U_{2}$ belonging to the concept pair $W_{1},W_{2}$, there is also a difference. $U_{}^{{\bar{W}}_{1},W_{2}}$ represents the set of instances that belong only to concept $W_{2}$. $U_{}^{W_{1},{\bar{W}}_{2}}$ represents the set of instances that belong only to concept $W_{1}$. *U*^*W*_1_,*W*_2_^ does not belong to the set of instances of the concept pair *W*_1_, *W*_2_.

Then we can denote the computational representation of $Sim_{ins}(W_{1},W_{2})$ based on the relationship between the two instance sets.

(5) $$Sim_{ins}(W_{1},W_{2})=\left\{ \begin{array}{l} Ins_{rich}\times Sim_{jac}(W_{1},W_{2}),Ins_{con}\leq T\\ Ins_{rich}\times \frac{U^{W_{1}}\cap U^{W_{2}}}{2\times Min(\left|U^{W_{1}}\right|,\left|U^{W_{2}}\right|)},Ins_{con}>T \end{array}\right..$$

$Ins_{rich}$ is the richness of the instance collection. $Ins_{con}$ is the instance set contrast value, $Sim_{jac}(W_{1},W_{2})$ is Jaccard’s similarity, which is used to express the similarity of concepts to $W_{1},W_{2}$. T is the threshold for the contrast of the set of instances.

By designing the richness of the instance collection $Ins_{rich}$, we can consider the specific differences of the collection of concept instances based on the Jaccard method to reduce the inaccuracy of similar results.

The definition of $Ins_{rich}$ in our strategy is as follows:

(6) $$Ins_{rich}=\frac{\log(\left|U_{1}\right|)+\log(\left|U_{2}\right|)}{2*\log(\left|U_{1}\right|)\cdot \log(\left|U_{2}\right|)+\eta_{rich}}.$$

For the case where the denominator may be 0, the parameter $\eta_{rich}$ is set in the formula. The richness of the set of instances is judged by the above formula. When the richness of an instance set is greater, the concept instance’s similarity is higher.

In addition, we also set $Ins_{con}$ to reflect the degree of difference in the number of instances. In the case of a large difference in the number of instances, $U_{1}\in \phi ,U_{2}\in \phi$ may occur. In order to calculate the similarity in this case, we use the value of $Ins_{con}$ to select different similarity calculation strategies.

The contrast of the $Ins_{con}$ instance set is denoted as follows:

(7) $$Ins_{con}=\frac{1}{\pi }(atan(\frac{\left|U_{1}^{}\right|}{\left|U_{2}^{}\right|+\eta_{con}})+atan(\frac{\left|U_{2}^{}\right|}{\left|U_{1}^{}\right|+\eta_{con}})).$$

Based on the richness of the instance set and the contrast of the instance set, we improved the Jaccard similarity calculation. As shown in the calculation formula of $Sim_{ins}(W_{1},W_{2})$ above, we use $Ins_{rich}$ as the coefficient of Jaccard similarity calculation. When $U_{1}\in \phi ,U_{2}\in \phi$ appears in the instance set, the similarity calculation form among the instance sets is adjusted to the ratio of the intersection of the instance set $U_{1},U_{2}$. The set of calculation instances is adjusted to twice the number of instances in $\left|U^{W_{1}}\right|,\left|U^{W_{2}}\right|$. The new similarity calculation formulae are as follows:

(8) $$Sim_{jac}(W_{1},W_{2})=\frac{P(W_{1},W_{2})}{P(W_{1},W_{2})+P(\bar{W_{1}},W_{2})+P(W_{1},\bar{W_{2}})}.$$

(9) $$P(W_{1},W_{2})=\frac{\left|U_{1}^{W_{1},W_{2}}\right|+\left|U_{2}^{W_{1},W_{2}}\right|}{\left|U_{1}^{}\right|+\left|U_{2}^{}\right|}.$$

(10) $$P(\bar{W_{1}},W_{2})=\frac{\left|U_{1}^{\bar{W_{1}},W_{2}}\right|+\left|U_{2}^{\bar{W_{1}},W_{2}}\right|}{\left|U_{1}^{}\right|+\left|U_{2}^{}\right|}.$$

(11) $$P(W_{1},\bar{W_{2}})=\frac{\left|U_{1}^{W_{1},{\bar{W}}_{2}}\right|+\left|U_{2}^{W_{1},\bar{W_{2}}}\right|}{\left|U_{1}^{}\right|+\left|U_{2}^{}\right|}.$$

When calculating the Jaccard similarity, we need to adopt a strategy to divide the sample set $U_{1},U_{2}$ of concept pair $W_{1},W_{2}$ into positive and negative samples. Due to the large number of sample instances, it is not practical for this strategy to be performed manually. However, by collecting part of the actual sample data and tag set, we can use machine learning classification algorithms to carry out this huge workload. In our method, a random forest algorithm has a good tolerance on the continuous and discrete attribute values of the sensor attributes. At the same time, a random forest algorithm has an excellent classification effect under supervised learning.

### 4.3. Structural Strategy

Concept is one of the elements of ontology, its information corresponding to the structure of the ontology. It can also be regarded as a semantic level in its hosting ontology. Based on the structural information of the ontology, we can calculate the degree of similarity between concepts from a new level.

First of all, we need to build the ontology tree based on the ontology structure diagram. For the two isomerism ontology trees, the similarity relationship between ontology concepts can be transformed into the similarity between two concept nodes in the ontology tree. By setting a similar search radius, *r*, which has a value of 3, 5, 7 for instance, a set of concepts on the ontology tree within a certain search range can be constructed. On the two ontology trees of isomerism, the same operation is performed on the calculation elements, and two related concept collections N(W) are constructed.

Structural similarity calculation rules are as follows:

1. In the constructed ontology tree, we assume that the uncles of the parent nodes of the two concepts are similar, and we believe that the two concepts are similar;
2. In the case where the two concept nodes are similar, their respective child nodes are also similar;
3. In the case where the two concept nodes are similar, their respective siblings are also similar.

According to the above rules, we use Jaccard’s coefficient to describe the similarity relationship between the two sets. The structure-based structural similarity calculation is denoted as follows:

(12) $$Sim_{str}(W_{1},W_{2})=\frac{\sigma Sim_{u}(W_{1},W_{2})+\tau Sim_{s}(W_{1},W_{2})+\upsilon Sim_{b}(W_{1},W_{2})}{\sigma +\tau +\upsilon }.$$

(13) $$Sim_{u}(W_{1},W_{2})=Sim_{jac}(N_{u}\left(W_{1}\right),N_{u}\left(W_{2}\right)).$$

(14) $$Sim_{s}(W_{1},W_{2})=Sim_{jac}(N_{s}\left(W_{1}\right),N_{s}\left(W_{2}\right)).$$

(15) $$Sim_{b}(W_{1},W_{2})=Sim_{jac}(N_{b}\left(W_{1}\right),N_{b}\left(W_{2}\right)).$$

(16) $$Sim_{jar}(N\left(W_{1}\right),N\left(W_{2}\right))=\frac{P(N\left(W_{1}\right),N\left(W_{2}\right))}{P(N\left(W_{1}\right),N\left(W_{2}\right))+P(\bar{N\left(W_{1}\right)},N\left(W_{2}\right))+P(N\left(W_{1}\right),\bar{N\left(W_{2}\right)})}.$$

where $W_{1},W_{2}$ represent the concept in two ontologies. $Sim_{u}(W_{1},W_{2})$ represents the similarity among the set of uncle nodes of parent nodes of $W_{1},W_{2}$. $Sim_{s}(W_{1},W_{2})$ represents the similarity among the set of child nodes of parent nodes of $W_{1},W_{2}$. $Sim_{b}(W_{1},W_{2})$ represents the similarity among the set of sibling nodes of parent nodes of $W_{1},W_{2}$. We consider different degrees of influence on the calculation of the overall structural similarity among the uncles, children, and siblings of the node. Set σ,τ,υ to indicate different influence coefficients, and σ+τ+υ=1, σ≥τ≥υ. The range of values of σ highlights the effect of the element’s uncle nodes on the overall similarity calculation.

N(W) represents the collection of nodes related concept W. Based on this, we add u,s,b to represent the set of uncles, children, and siblings of the parent. The elements in these collections are all concepts in the ontology.

In addition, we also consider that the ontology tree constructed by different search radius r has a different influence on the calculation of similarity. Thus, we revise the calculation method for structural similarity as follows:

(17) $$Sim_{str,r}(W_{1},W_{2})=\frac{\sigma_{r}Sim_{u}(W_{1},W_{2})+\tau_{r}Sim_{s}(W_{1},W_{2})+\upsilon_{r}Sim_{b}(W_{1},W_{2})}{\sigma_{r}+\tau_{r}+\upsilon_{r}}.$$

(18) $$Sim_{str}(W_{1},W_{2})=\frac{\sum_{r}Sim_{str,r}(W_{1},W_{2})}{num(r)}.$$

According to different degrees of influence, we use $\sigma_{r},\tau_{r},\upsilon_{r}$ to represent coefficients that differ according to the search radius r and num(r) indicates the set number of searches.

### 4.4. Ontology Mapping Rules

Without loss of generality, for two ontologies *O*_1_ and *O*_2_, assume that there are m concepts in the ontology $O_{1}$ to be mapped and there are n concepts in the ontology $O_{2}$ to be mapped. Then the result of mapping between ontologies is a m×n matrix Mat. We use Mat[i][j],i∈[0,m−1],j∈[0,n−1] to indicate the degree of similarity between the ith concept in the ontology $O_{1}$ to be mapped and the jth concept in the ontology $O_{2}$ to be mapped.

According to our previously concept-based similarity calculation strategy, we can calculate each value in matrix Mat. However, in the actual process, we cannot directly find out the set of the most similar concept pairs in the matrix as the result of ontology mapping. Since the similarity computation of the three different strategies have respective emphases and require an unequal computation load, we can use an analytic hierarchy process (AHP) to optimize the similarity calculation as shown in Algorithm 5.


**Algorithm 5 AHP algorithm**

Initialize the m×n matrix Mat through semantic similarity computation.Set different thresholds $T_{sem}$, $T_{ins},T_{str}$ for three different strategies.For T = {$T_{sem}$, $T_{ins},T_{str}$}:  For i,j. i∈[0,m−1],j∈[0,n−1]:    If Mat[i][j]<T:     Mat[i][j]=0.    else     Mat[i][j] retains the original value.For i. i∈[0,m−1]: For j. j∈[0,n−1]:     find the largest Mat[i][j] in each row.After finding the matrix Mat, we can get all mapping rules based on concept pairs between ontology $O_{1},O_{2}$.


In this AHP based similarity calculation, we can initialize the matrix by computing similarity based on semantic strategy. This takes precedence over other similarity calculation strategies, as the similarity degree based on semantics can effectively exclude some concept pairs with low correlation between ontologies. Thus, the subsequent similarity computation only needs to be done in the concept pairs that we are interested in. In this process, we can obtain the final ontology mapping matrix by using the three different similarity calculation strategies.

By setting threshold parameters $T_{sem},T_{ins},T_{str}$ and scanning the final ontology mapping matrix, we can determine that concept i in the ontology $O_{1}$ is associated with concept j in the ontology $O_{2}$.

## 5. Experimental Results

In order to verify that our method is effective in the practical application of ontology correlation, we introduce the experimental results of the case study of semantic inference for berth management. We use the sensors registered by 52North [50] to get the depth conditions and climatic conditions of port berths. Data generated by our simulator is also used to test our proposed method. Through semantic mapping, we transform the sensor data in the database into instances of SSN ontology and store them in OWL files. In order to extract the concepts and attributes corresponding to the sensor data in the SSN ontology and make the database model corresponding to the SSN ontology model, we use the following XML mapping language pattern. The corresponding elements of sensor data are shown in the following Table 1 where the concept of sensors is mapped to the SSN/SOSA ontology framework [51], respectively. When building the SSN ontology instances, we denote the corresponding relationship between the elements in the mapping language and classes in the SSN ontology. For different types of sensors, we generate the SSN ontology instances based on the 52North real sensor data and the corresponding sensor data from our simulator. As to the establishment of domain ontology, we focus on the analysis of the various aspects of the port monitoring.

A semi-automated domain ontology construction method is adopted with expert opinions in order to build two ontologies: the ship berth management ontology and the port monitoring ontology, which are designed to provide support for the port administration to grasp real-time information and make appropriate operation decisions.

The ship berth management ontology is used to analyze the changes of ship berth scheduling, entry and exit berth, hydrology, weather and other related data, making timely decisions according to the corresponding berth management plan. In general, the ship berth management makes a corresponding plan according to the different levels judged by the comprehensive situation of the ship berth. The ship berth management ontology contains a number of concepts about various aspects of berths under different scenarios, and its brief structure is shown in the Table 2. The port monitoring ontology is an ontology that contains comprehensive information of the port which has the goal to achieve fully automated operations. This ontology mainly includes ship management, container management, port cargo handling management, port hydrological management and many other objectives. Among them, port hydrology management also contains many concepts about water environment for a port. The examples under these concepts are built on the basis of a large number of sensors in a port. The brief structure of this domain ontology for port monitoring is shown in the Table 3.

Based on the above description of the experimental domain ontologies, we partially select and test seven concepts in the ship berth management ontology and nine concepts in the port monitoring ontology to test our concept-based similarity calculation method between ontologies. The definition of each concept consists of 6 parts: concept name, concept instance set, concept semantic neighbor set, concept composition, function and attribute set.

Conceptual instance sets are used to calculate similarity based on conceptual instances; concept names and attribute set are used together to calculate semantic similarity; the semantic neighbor set of the concept is used to calculate structural similarity. The set of attributes for each concept is the union of all the different kinds of sensor attributes we have collected. This operation provides a unified representation of sensor data.

**Figure 1:**
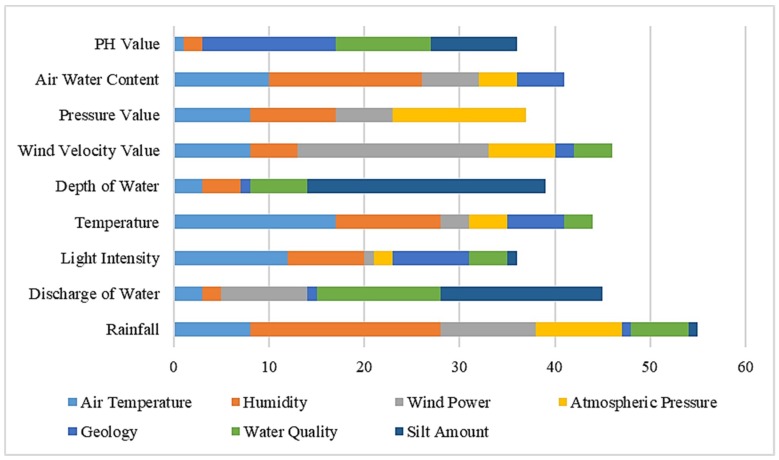
Attribute intersection distribution of random forest division.

For the sensor instance, the different attribute set distributions are shown in the following Table 4. The total represents the number of instances contained under each concept name, that is, the size of the concept instance set. Similarly, we classify the concept of instances according to the values of each attribute set according to the random forest algorithm. Figure 1 and Table 4 reflect the distribution of the conceptual instances. RF, DIW, LI, T, DEW, WVV, PV, AWC, PHV, AT, H, WP, AP, G, WQ, SA are the abbreviations of Rainfall, Discharge of Water, Light Intensity, Temperature, Depth of Water, Wind Velocity Value, Pressure Value, Air Water Content, PH Value, Air Temperature, Humidity, Wind Power, Atmospheric Pressure, Geology, Water Quality, Silt Amount respectively.

As shown in Table 4, we can see that the concept instance set size of the RF is 55, and the instance with a size of 8 can also be used as part of the concept instance set of AT. This is the case because the concept of rainfall has a certain overlapping relationship with the concept of temperature. Therefore, according to the data collected by the sensors on each attribute, there is also an intersection part of their instance sets.

**Figure 2:**
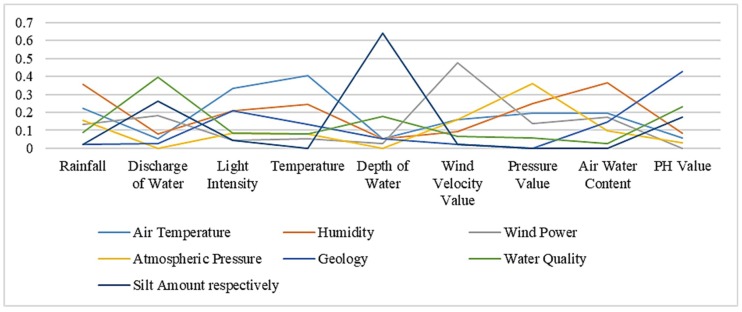
Similarity results based on instance strategy.

**Figure 3:**
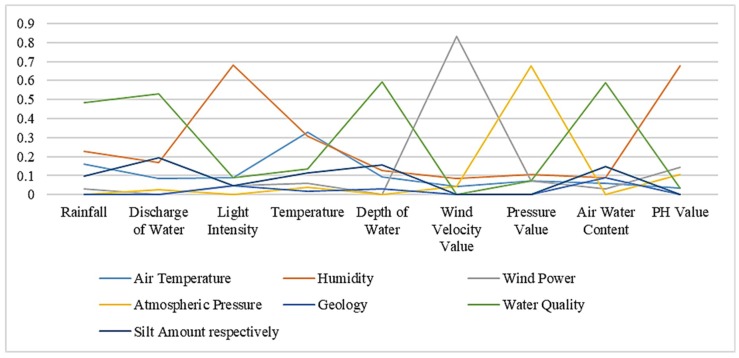
Similarity results based on semantic strategy.

**Figure 4:**
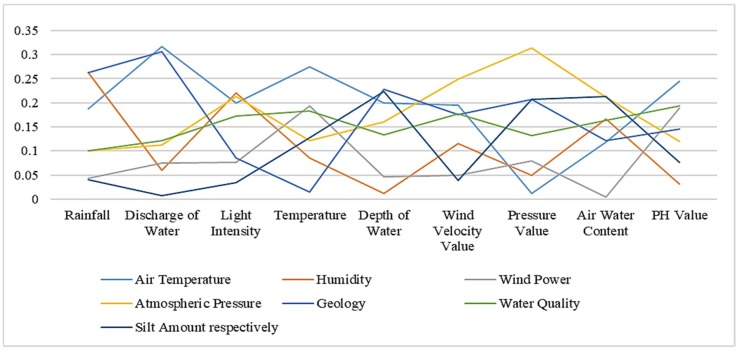
Similarity results based on structural strategy.

Using the similarity computation method based on instance strategy, we get the similarity between the two ontologies in the following Table 5. Table 6 and Table 7 respectively represent similarity results based on semantic strategy and results based on structural strategy. The bold numbers in the tables indicate the highest value in each column. Figure 2, Figure 3 and Figure 4 show similarity calculation results in a more intuitive form.

Different from other synthetic methods of similarity computation, we use the analytic hierarchy process (AHP) and three different strategies to screen similarity. In this experiment, first we eliminate concept pairs below the threshold by using a semantic-based similarity strategy. Assuming a threshold of 0.03, concept pairs like geology-rainfall, atmospheric pressure-rainfall, wind power-discharge of water, geology-discharge of water can be eliminated. Then we set the threshold to 0.04 and further filter based on structural strategy. Finally, by setting the threshold to 0.2, we can use the instance-based similarity strategy to get the result of the concept match. 

**Figure 5:**
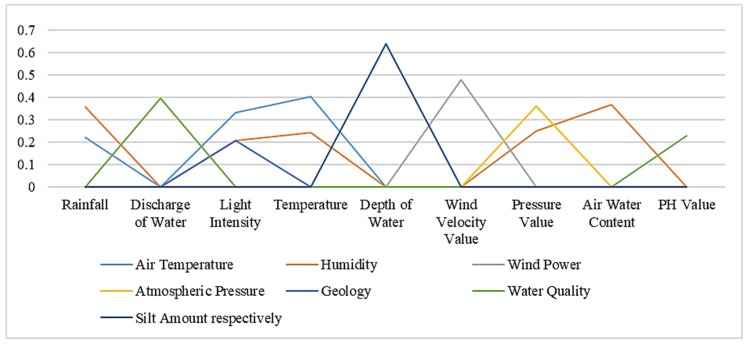
Similarity results based on analytic hierarchy process (AHP).

As shown in Figure 5 and Table 8. This method, on the one hand, eliminates the need for domain experts to adjust the weight of the comprehensive calculation. On the other hand, it reduces the calculation consumption of some unnecessary concepts.

According to the results shown in Table 8, where the bold numbers in the table indicate the highest value in each column, we use the AHP method to set the screening thresholds for each level, and the results of the three similarity calculation strategies mentioned in this paper are processed hierarchically. As can be seen from the data in the above table, after the multi-strategy similarity evaluation, some concept pairs have strong similarities, such as RF-H, DIW-WQ, LI-AT, and the like. At the same time, the weaker similarity between most conceptual pairs is reduced to zero. After processing the data shown in Table 8 with the computations of steps 4 and 5 in the AHP algorithm shown in Algorithm 5, we use the concept pairs with the largest similarity as the mapping relationship of the corresponding concepts in the ontologies. 

In terms of the domain ontologies we evaluated in this experiment—ship berth management ontology and port monitoring ontology—the experimental results of our ontology mapping can effectively help to link multiple ontologies, thus achieving the linkage between port monitoring and ship berth management. This intelligent linkage is very useful in real-world autonomous industrial operations such as maintenance work for ship berths in our experiment. By monitoring the port hydrology in real time through many sensors and mapping the hydrological monitoring data to the ship berth management system, effective ship berth maintenance can be achieved by a multi-level management plan and a rule reasoning library.

In addition, in order to further evaluate our proposed method, we also utilize the ontology mapping calculation strategies proposed in the other four ontology mapping systems (Rimom [52], ASMOV [53], Falcon [54] and OntoDNA [55]) in our experimental system too. We evaluate the performance of our method by conducting two sets of experiments. We first use all these five strategies to perform the ontologies’ correlation task with the sensor data and two domain ontologies that are used in our simulation experiment; the experimental results are shown in Table 9. As our method uses sensor data to generate sensor instances to increase the instance set size of the ontology concept, the degree of similarity between concepts in the perspective of instance collection can be measured. This makes our method superior to others. It can be seen from the experimental results that, compared with other ontology mapping methods, our method has achieved relatively better results in term of recall (Rec.), precision (Pre.) and F-measure (F.), whose calculation method is defined as the following formulae in [19,20]. In the evaluation, we divide the prediction result into four cases (true positive, true positive, false positive and false negative). In the formula, true positive, true negative, false positive, and false negative represent the specific values in different cases.

(19) $$recall\left(Rec.\right)=\frac{TruePositive}{TruePositive+FalsePositive}.$$

(20) $$precision\left(Pre.\right)=\frac{TruePositive}{TruePositive+FalseNegative}.$$

(21) $$F-measure\left(F.\right)=\frac{2\times recall(Rec.)\times precision(Pre.)}{recall(Rec.)+precision(Pre.)}.$$

In terms of ontology mapping performance, another set of experiments is conducted to evaluate the effectiveness of the similarity computation strategy between ontology concepts that we proposed in Section 4. We compare our methods again with the four ontology mapping calculation strategies of other mapping systems (Rimom, ASMOV, Falcon, OntoDNA). These experiments used the ontologies numbered as #101–#304 in the OAEI standard test dataset benchmarks [56] as the evaluation target. Among them, ontology #101 is used as reference ontology, #1XX represents all special ontologies, #2XX represents all ontologies lacking semantic information in some aspects, and #3XX represents all actual ontologies. As shown in Table 10, where the bold numbers in the table indicate the highest value in each column, the results of the ontology mapping experiment are evaluated in terms of the recall (Rec.), precision (Pre.) and F-measure (F.), which are defined above. It can be seen from the experimental results that the multi-strategy similarity calculation method of this paper can achieve almost all mapping relationships in the OAEI data set.

## 6. Conclusions

Associating sensor data with existing domain ontologies is an effective way to give richer semantic meaning to the sensor data, and to realize the sharing, reuse and fusion of that sensor data. In this work, we have proposed a mechanism to associate sensor data with multiple domain ontologies. Our mechanism uses a random forest-based learning model to classify the sensor instances, thus greatly reducing the workload of manual analysis and labeling. In the meantime, by adding the classified sensor instances into the instance set of specific concepts of the ontology, the instance set can be effectively expanded. Based on this, a novel multi-strategy method is proposed to construct the ontology relations, and we use the analytic hierarchy process to analyze the similarity of concept pairs in ontologies. Through the calculation of semantic similarity, instance similarity, and structural similarity, we associate the concept pairs with high similarity in the ontologies, and finally establish the mapping of sensor data and multiple domain ontologies. In future work, we will continue to optimize the strategy for associating sensor data with ontologies to make better use of the enormous heterogenous sensor data.

## Figures and Tables

**Table 1:** Sensor Element Mapping.

Sensor Element mapping	Source mapping	Table name	Sensor_num	Sensor number	sosa:madeBySensor
			Observationvalue	Collection of sensor observation value	sosa:hasResult
			Observationtime	Time of observation data	sosa:resultTime
		Source_id		Selected data source number	sosa:observedProperty
	Data mapping	Source_id		Data source number	sosa:observedProperty
		Sensor_id		Number of sensor instances	sosa:madeBySensor
		Type		Sensor type	sosa:observes
		Unit		Observational unit	sosa:Result
		Location_name		Sensor position	ssn:hasDeployment

**Table 2:** The Ship Berth Management Ontology.

Ship Berth Management	Multi-level regular plan management	First-level plan	Next concept list: Air Temperature, Humidity, Wind Power, Atmospheric Pressure, Geology, Water Quality, Silt Amount, etc.
		Second-level plan	Next concept list: Air Temperature, Humidity, Wind Power, Atmospheric Pressure, Geology, Water Quality, Silt Amount, etc.
		Three-level plan	Next concept list: Air Temperature, Humidity, Wind Power, Atmospheric Pressure, Geology, Water Quality, Silt Amount, etc.
		… …	… …
	Emergency plan management	Emergency situations I	… …
		Emergency situations II	… …
		… …	… …

**Table 3:** The Port Monitoring Ontology.

Port Monitoring	ship management	… …	
	container management	… …	
	port cargo handling management	… …	
	port hydrological management	Class A water environment	Next concept list: Rainfall, Discharge of Water, Light Intensity, Temperature, Depth of Water, Wind Velocity Value, Pressure Value, Air Water Content, PH Value, etc.
		Class B water environment	Next concept list: Rainfall, Discharge of Water, Light Intensity, Temperature, Depth of Water, Wind Velocity Value, Pressure Value, Air Water Content, PH Value, etc.
		Class C water environment	Next concept list: Rainfall, Discharge of Water, Light Intensity, Temperature, Depth of Water, Wind Velocity Value, Pressure Value, Air Water Content, PH Value, etc.
		… …	… …
	… …	… …	

**Table 4:** Attribute intersection distribution of random forest division.

	RF	DIW	LI	T	DEW	WVV	PV	AWC	PHV	Total
**AT**	8	3	12	17	3	8	8	10	1	70
**H**	20	2	8	11	4	5	9	16	2	77
**WP**	10	9	1	3	0	20	6	6	0	55
**AP**	9	0	2	4	0	7	14	4	0	40
**G**	1	1	8	6	1	2	0	5	14	38
**WQ**	6	13	4	3	6	4	0	0	10	46
**SA**	1	17	1	0	25	0	0	0	9	53
**Total**	55	45	36	44	39	46	37	41	36	

**Table 5:** Similarity results based on instance strategy.

	RF	DIW	LI	T	DEW	WVV	PV	AWC	PHV
**AT**	0.222	0.053	**0.333**	**0.405**	0.051	0.159	0.194	0.195	0.057
**H**	**0.356**	0.079	0.208	0.243	0.051	0.091	0.250	**0.366**	0.086
**WP**	0.133	0.184	0.042	0.054	0.026	**0.477**	0.139	0.171	0
**AP**	0.156	0	0.083	0.081	0	0.159	**0.361**	0.098	0.029
**G**	0.022	0.026	0.208	0.135	0.051	0.023	0	0.146	**0.429**
**WQ**	0.089	**0.395**	0.083	0.081	0.179	0.068	0.056	0.024	0.229
**SA**	0.022	0.263	0.042	0	**0.641**	0.023	0	0	0.171

**Table 6:** Similarity results based on semantic strategy.

	RF	DIW	LI	T	DEW	WVV	PV	AWC	PHV
**AT**	0.161	0.083	0.091	**0.327**	0.094	0.042	0.071	0.059	0.036
**H**	0.226	0.167	**0.682**	0.308	0.125	0.083	0.107	0.088	**0.679**
**WP**	0.032	0	0.045	0.058	0	**0.833**	0.071	0.029	0.143
**AP**	0	0.028	0	0.038	0	0.042	**0.679**	0	0.107
**G**	0	0	0.045	0.019	0.031	0	0	0.088	0
**WQ**	**0.484**	**0.528**	0.091	0.135	**0.594**	0	0.071	**0.588**	0.036
**SA**	0.097	0.194	0.045	0.115	0.156	0	0	0.147	0

**Table 7:** Similarity results based on structural strategy.

	RF	DIW	LI	T	DEW	WVV	PV	AWC	PHV
**AT**	0.187	**0.317**	0.200	**0.274**	0.199	0.195	0.012	0.119	**0.245**
**H**	**0.263**	0.060	**0.220**	0.086	0.012	0.116	0.050	0.166	0.031
**WP**	0.044	0.075	0.076	0.193	0.046	0.049	0.079	0.005	0.189
**AP**	0.101	0.113	0.213	0.122	0.160	**0.249**	**0.314**	0.211	0.120
**G**	**0.263**	0.306	0.085	0.015	**0.228**	0.175	0.207	0.122	0.145
**WQ**	0.101	0.121	0.172	0.183	0.133	0.177	0.132	0.164	0.194
**SA**	0.041	0.008	0.034	0.127	0.223	0.039	0.207	**0.213**	0.077

**Table 8:** Similarity results based on structural strategy.

	RF	DIW	LI	T	DEW	WVV	PV	AWC	PHV
**AT**	0.222	0	**0.333**	**0.405**	0	0	0	0	0
**H**	**0.356**	0	0.208	0.243	0	0	0.25	**0.366**	0
**WP**	0	0	0	0	0	**0.477**	0	0	0
**AP**	0	0	0	0	0	0	**0.361**	0	0
**G**	0	0	0.208	0	0	0	0	0	0
**WQ**	0	**0.395**	0	0	0	0	0	0	**0.229**
**SA**	0	0	0	0	**0.641**	0	0	0	0

**Table 9:** Experimental comparison with other methods.

	Rec.	Pre.	F.
**Rimom**	0.85	0.94	0.893
**ASMOV**	0.82	0.87	0.844
**Falcon**	0.76	0.91	0.828
**OntoDNA**	0.77	0.88	0.821
**This paper**	**0.85**	**0.95**	**0.897**

**Table 10:** Experimental results based on OAEI data set.

	Rec.			Pre.			F.		
	#1XX	#2XX	#3XX	#1XX	#2XX	#3XX	#1XX	#2XX	#3XX
Rimom	1.00	0.79	**0.87**	0.99	**0.97**	**0.96**	**0.995**	0.871	**0.913**
ASMOV	1.00	0.84	0.85	0.98	0.88	0.71	0.989	0.860	0.774
Falcon	1.00	0.86	0.79	0.98	0.96	0.87	0.989	0.907	0.828
OntoDNA	1.00	0.76	0.78	0.97	0.78	0.94	0.985	0.770	0.853
This paper	**1.00**	**0.87**	0.85	**0.99**	0.95	0.88	**0.995**	**0.908**	0.865

## References

[1] Wang J., Gao Y., Yin X., Li F., Kim H.J. (2018). An Enhanced PEGASIS Algorithm with Mobile Sink Support for Wireless Sensor Networks. Wirel. Commun. Mob. Comput..

[2] Li X., Niu J., Kumari S., Wu F., Sangaiah A.K., Choo K.K.R. (2018). A Three-factor Anonymous Authentication Scheme for Wireless Sensor Networks in Internet of Things Environments. J. Netw. Comput. Appl..

[3] Wang J., Cao J., Sherratt R.S., Park J.H. (2018). An improved ant colony optimization-based approach with mobile sink for wireless sensor networks. J. Supercomput..

[4] Tirkolaee E., Hosseinabadi A., Soltani M., Sangaiah A., Wang J. (2018). A hybrid genetic algorithm for multi-trip green capacitated arc routing problem in the scope of urban services. Sustainability.

[5] Lin F., Zhou Y., An X., You I., Choo K.K.R. (2018). Fair Resource Allocation in an Intrusion-Detection System for Edge Computing: Ensuring the Security of Internet of Things Devices. IEEE Consum. Electron. Mag..

[6] Wan S., Zhao Y., Wang T., Gu Z., Abbasi Q.H., Choo K.K.R. (2019). Multi-dimensional data indexing and range query processing via Voronoi diagram for internet of things. Future Gener. Comput. Syst..

[7] Eid M., Liscano R., El S.A. A universal ontology for sensor networks data. Proceedings of the 2007 IEEE International Conference on Computational Intelligence for Measurement Systems and Applications.

[8] Khaleghi B., Khamis A., Karray F.O., Razavi S.N. (2013). Multisensor data fusion: A review of the state-of-the-art. Inf. Fusion.

[9] Wang J., Zhang Z., Li B., Lee S.Y., Sherratt R.S. (2014). An Enhanced Fall Detection System for Elderly Person Monitoring using Consumer Home Networks. IEEE Trans. Consum. Electron..

[10] Compton M., Barnaghi P., Bermudez L., GarcíA-Castro R., Corcho O., Cox S., Graybeal J., Hauswirth M., Henson C., Herzog A. (2012). The SSN ontology of the W3C semantic sensor network incubator group. Web Semant. Sci. Serv. Agents World Wide Web.

[11] Compton M., Henson C., Lefort L., Neuhaus H., Sheth A.P. A survey of the semantic specification of sensors. Proceedings of the International Conference on Semantic Sensor Networks.

[12] Wang J., Ju C., Gao Y., Sangaiah A.K., Kim G.J. (2018). A PSO based energy efficient coverage control algorithm for wireless sensor networks. Comput. Mater. Contin..

[13] Sheng Z., Mahapatra C., Leung V.C.M., Chen M., Sahu P.K. (2018). Energy efficient cooperative computing in mobile wireless sensor networks. IEEE Trans. Cloud Comput..

[14] Wang J., Gao Y., Liu W., Sangaiah A.K., Kim H.J. (2019). An Improved Routing Schema with Special Clustering Using PSO Algorithm for Heterogeneous Wireless Sensor Network. Sensors.

[15] Li Z., Xia C., Zhang C. (2013). Research on Information Sharing of Sensor Networks Based on Ontology. Sci. Technol. Vis..

[16] Liu J., Zhou M., Lin L., Kim H.J., Wang J. (2018). Rank web documents based on multi-domain ontology. J. Ambient Intell. Humaniz. Comput..

[17] Ehrig M., Sure Y. (2004). Ontology Mapping-An Integrated Approach. European Semantic Web Symposium.

[18] Mao M., Peng Y., Spring M. (2010). An adaptive ontology mapping approach with neural network based constraint satisfaction. Web Semant. Sci. Serv. Agents World Wide Web.

[19] Otero-Cerdeira L., Rodríguez-Martínez F.J., Gómez-Rodríguez A. (2014). Definition of an ontology matching algorithm for context integration in smart cities. Sensors.

[20] Fernandez S., Marsa-Maestre I., Velasco J.R., Alarcos B. (2013). Ontology alignment architecture for semantic sensor web integration. Sensors.

[21] Doan A.H., Madhavan J., Domingos P., Halevy A. (2004). Ontology matching: A machine learning approach. Handbook on Ontologies.

[22] Juan Y.U. (2008). Review on Ontology Integration. Comput. Sci..

[23] Fan L., Wang A., Xiao Y. (2007). Research on Evaluation Index System of Ontology Integration Method and Its Application. Comput. Integr. Manuf. Syst..

[24] Krishnan K., Krishnan R., Muthumari A. (2017). A semantic-based ontology mapping–information retrieval for mobile learning resources. Int. J. Comput. Appl..

[25] Zeng D., Dai Y., Li F., Sherratt R.S., Wang J. (2018). Adversarial Learning for Distant Supervised Relation Extraction. CMC Comput. Mater. Contin..

[26] Tu Y., Lin Y., Wang J., Kim J.U. (2018). Semi-supervised Learning with Generative Adversarial Networks on Digital Signal Modulation Classification. Comput. Mater. Contin..

[27] Hooi Y.K., Hassan M.F., Shariff A.M. (2014). A Survey on Ontology Mapping Techniques. Computer Science and Its Applications.

[28] Ma Z., Zhang F., Yan L., Cheng J. (2014). Fuzzy Semantic Web Ontology Mapping. Fuzzy Knowledge Management for the Semantic Web.

[29] Jung M., Jun H.B., Kim K.W., Suh H.W. (2010). Ontology mapping-based search with multidimensional similarity and Bayesian network. Int. J. Adv. Manuf. Technol..

[30] Swat M.J., Grenon P., Wimalaratne S. (2016). ProbOnto: Ontology and knowledge base of probability distributions. Bioinformatics.

[31] Moran N., Nieland S., Kleinschmit B. (2017). Combining machine learning and ontological data handling for multi-source classification of nature conservation areas. Int. J. Appl. Earth Obs. Geoinf..

[32] Ravikumar G., Vijayan S. (2017). A Machine Learning Approach for MRI Brain Tumor Classification. CMC Comput. Mater. Contin..

[33] Liu J., Ren H., Wu M., Wang J., Kim H.J. (2018). Multiple relations extraction among multiple entities in unstructured text. Soft Comput..

[34] Amrouch S., Mostefai S. Survey on the literature of ontology mapping, alignment and merging. Proceedings of the IEEE International Conference on Information Technology and e-Services (ICITeS).

[35] Du J., Sugumaran V. Ontology-Based Information Integration and Decision Making in Prefabricated Construction Component Supply Chain. Proceedings of the Semantics, Ontologies, Intelligence and Intelligent Systems (SIGODIS).

[36] Caldarola E.G., Picariello A., Rinaldi A.M. (2015). An approach to ontology integration for ontology reuse in knowledge based digital ecosystems. Proceedings of the 7th International Conference on Management of Computational and Collective intElligence in Digital EcoSystems.

[37] Khattak A.M., Pervez Z., Khan W.A., Khan A.M., Latif K., Lee S.Y. (2015). Mapping evolution of dynamic web ontologies. Inf. Sci..

[38] Chaabane S., Jaziri W. (2018). A novel algorithm for fully automated mapping of geospatial ontologies. J. Geogr. Syst..

[39] Arnold P., Rahm E. (2014). Enriching ontology mappings with semantic relations. Data Knowl. Eng..

[40] Wang M., Wang J., Guo L., Harn L. (2018). Inverted XML Access Control Model Based on Ontology Semantic Dependency. Comput. Mater. Contin..

[41] Xiong Z., Shen Q., Wang Y., Zhu C. (2018). Paragraph Vector Representation Based on Word to Vector and CNN Learning. CMC Comput. Mater. Contin..

[42] Mikolov T., Le Q.V., Sutskever I. (2013). Exploiting similarities among languages for machine translation. arXiv.

[43] Gao W., Farahani M.R., Aslam A., Hosamani S. (2017). Distance learning techniques for ontology similarity measuring and ontology mapping. Clust. Comput..

[44] Dou D., Wang H., Liu H. (2015). Semantic data mining: A survey of ontology-based approaches. Proceedings of the 2015 IEEE International Conference on Semantic Computing (ICSC).

[45] Bytyçi E., Ahmedi L., Lisi F.A. (2017). Enrichment of Association Rules through Exploitation of Ontology Properties–Healthcare Case Study. Procedia Comput. Sci..

[46] Pinkel C., Binnig C., Jiménez-Ruiz E., Kharlamov E., May W., Nikolov A., Sasa Bastinos A., Skjæveland M.G., Solimando A., Taheriyan M. (2016). RODI: Benchmarking relational-to-ontology mapping generation quality. Semant. Web.

[47] Forsati R., Shamsfard M. (2016). Symbiosis of evolutionary and combinatorial ontology mapping approaches. Inf. Sci..

[48] Helou M.A., Palmonari M., Jarrar M. (2016). Effectiveness of Automatic Translations for Cross-Lingual Ontology Mapping. J. Artif. Intell. Res..

[49] Zhang L., Yin C.Y., Chen J. (2010). Chinese word similarity computing based on semantic tree. J. Chin. Inf. Process..

[50] Henson C.A., Pschorr J.K., Sheth A.P., Thirunarayan K. (2009). SemSOS: Semantic sensor Observation Service. Proceedings of the IEEE International Symposium on Collaborative Technologies and Systems.

[51] Semantic Sensor Network Ontology. https://www.w3.org/TR/vocab-ssn/.

[52] Li Y., Li J.Z., Zhang D., Tang J. (2006). Result of Ontology Alignment with RiMOM at OAEI’06. Ontology Matching.

[53] Jean-Mary Y.R., Shironoshita E.P., Kabuka M.R. (2010). ASMOV: Results for OAEI 2010. Ontol. Matching.

[54] Hu W., Qu Y. (2008). Falcon-AO: A practical ontology matching system. Web Semant. Sci. Serv. Agents World Wide Web.

[55] Kiu C.C., Lee C.S. (2006). Ontology mapping and merging through OntoDNA for learning object reusability. Educ. Technol. Soc..

[56] Ontology Alignment Evaluation Initiative. http://oaei.ontologymatching.org/2007/benchmarks/.

